# Involving Latina/o parents in patient‐centered outcomes research: Contributions to research study design, implementation and outcomes

**DOI:** 10.1111/hex.12540

**Published:** 2017-02-08

**Authors:** Mónica Pérez Jolles, Maria Martinez, San Juanita Garcia, Gabriela L. Stein, Mentor Parent Group Members, Kathleen C. Thomas

**Affiliations:** ^1^ Suzanne Dworak‐Peck School of Social Work University of Southern California Los Angeles CA USA; ^2^ Psychology Department University of North Carolina at Greensboro Greensboro NC USA; ^3^ Cecil G. Sheps Center for Health Services Research University of North Carolina at Chapel Hill Chapel Hill NC USA; ^4^ Health Policy and Management University of North Carolina at Chapel Hill Chapel Hill NC USA

**Keywords:** collaborative research, Latinos/Hispanic, mental health, Patient Centered Outcomes Research

## Abstract

**Background:**

Comparative effectiveness research (CER) is supported by policymakers as a way to provide service providers and patients with evidence‐based information to make better health‐care decisions and ultimately improve services for patients. However, Latina/o patients are rarely involved as study advisors, and there is a lack of documentation on how their voices contribute to the research process when they are included as collaborators.

**Objectives:**

The purpose of this article was to contribute to the literature by presenting concrete contributions of Latina/o parent involvement to study design, implementation and outcomes in the context of a CER study called Padres Efectivos (Parent Activation).

**Methods:**

Researchers facilitated a collaborative relationship with parents by establishing a mentor parent group. The contributions of parent involvement in the following stages of the research process are described: (i) proposal development, (ii) implementation of protocols, (iii) analysis plan and (iv) dissemination of results.

**Results:**

Mentor parents’ contributions helped tailor the content of the intervention to their needs during proposal, increased recruitment, validated the main outcome measure and added two important outcome measures, emphasized the importance of controlling for novice treatment status and developed innovative dissemination strategies.

**Conclusions:**

Mentor parents’ guidance to the researchers has contributed to reaching recruitment goals, strengthened the study protocol, expanded findings, supported broad ownership of study implications and enriched the overall study data collection efforts. These findings can inform future research efforts seeking an active Latino parent collaboration and the timely incorporation of parent voices in each phase of the research process.

## 1 INTRODUCTION

Latinas/os are the largest and fastest growing minority population in the United States as one in four children are currently Latina/o, and by 2050, this trend will increase with estimations to about one in three.^1^ Despite national gains in positive health outcomes for Latina/o children in the past decade, there are persistent health‐care disparities in the use of needed mental health services.^2^, ^3^ Assessing the comparative effectiveness of interventions to overcome these disparities is a major national health research priority.^4^

Comparative effectiveness research (CER) is supported by policymakers as a way to provide service providers and patients with evidence‐based information to make better health‐care decisions and ultimately improve health outcomes f patients.^5^ However, there is a history of low patient involvement in CER studies among minority groups, especially among Latina/o patients.^6^ This low participation of Latina/os in CER research reduces the representativeness of these studies, the applicability of those results to the particular needs of this population and undermines ownership of study results.

While the impact of stakeholder engagement in research is an emerging field,^7^ the Patient‐Centered Outcomes Research Institute (PCORI) emphasizes the importance of stakeholder contributions in the research process to inform that process and increase the meaningfulness of findings. This “authentic engagement” involves stakeholders, such as patients and their caregivers, as active partners in all phases of research.^8^ This participatory process is also expected to reduce barriers to research participation among Latinas/os (e.g. mistrust, lack of health information and education, language barriers) as patient and caregiver advisors become more familiar with the research process and partner in decision making. There is evidence of growing efforts to include Latina/o patient voices in the research process. Latina/o participation as collaborators in research studies is documented in the literature in the areas of HIV prevention and mental health.^9^, ^10^, ^11^

Overlapping themes that frame the importance and impact of stakeholder engagement in the research process relate to participatory research, the quality of the research process and health disparities. That is, when researchers incorporate stakeholders’ own views on how to empower patients and their caregivers, own experiences with health services and their research priorities into research plans, implementation of protocols, translation of results and dissemination efforts, the hope is that this will help to (i) develop robust conceptual models, (ii) increase the relevance of researchers’ efforts, (iii) increase academic and stakeholder accountability, and legitimacy of findings in the eyes of stakeholders and communities.^5^ Patient and caregiver input also enhances the potential (iv) applicability and (v) uptake of findings because it allows researchers to address issues found important in patients’ own communities and in real time. Through this process, ideally researchers also build trust and respect as “reciprocal relationships” between stakeholders and researchers are developed.^7^, ^12^ This is critical in fostering a formal space, especially for historically under‐represented groups, to voice their opinions.

Despite the strong support and relevance for research quality, there is a dearth of information on the contributions of caregiver inclusion as collaborating stakeholders in the research process, especially in terms of research study design, implementation and outcomes. The purpose of this study is to contribute to the literature by describing concrete contributions of a Latina/o parent advisory group in the context of a PCORI‐funded CER study called Padres Efectivos (Parent Activation): Skills Latina Mothers Use to Get Healthcare For Their Children.^13^ The Padres Efectivos study was funded by the PCORI to test the impact of patient activation training tailored for Latina/o parents on parent and child outcomes. This organization requires active patient engagement in research as collaborators, and it supports this process through an engagement rubric (http://www.pcori.org/sites/default/files/Engagement-Rubric.pdf). We use the word “collaborative research” in this study to describe the role and contributions of our parents in the study as opposed to using the word “patient engagement” for the following reasons. First, our parents are not directly patients of the clinic but caregivers, and second, we follow PCORI's definition of patient engagement as “meaningful involvement of patients, caregivers, clinicians and other health‐care stakeholders throughout the research process—from topic selection through design and conduct of research to dissemination of results.”^14^

Patient activation is defined in this study as the role that an informed, engaged parent takes on as part of their child's health‐care team. Kinney et al.^15^ define patient activation as an individual's gained knowledge on available options for optimal health care and confidence to access needed resources. Activation also refers to a parent or patient's ability to be an active partner by self‐managing their own health and health care.^16^ Activation focuses on aspects of provider‐patient communication such as asking questions and getting what the patient perceives as needed out of the clinical encounter.^17^

This study presents a descriptive case study of the Padres Efectivos study to elucidate how Latino parent collaboration influenced our research study and to provide contextual information through the Padres Efectivos study.^18^ We chose a descriptive case study methodology because it allows us to describe the process of parents involved in research and how their contributions helped shape the research study proposal, design, implementation and outcomes. Our ultimate goal is that this study will inform similar efforts seeking to invite Latino parents as collaborators throughout the various phases of a research study.^19^ We describe the process and impacts of stakeholder engagement on five elements of the research process, (i) proposal development, (ii) implementation of protocols, (iii) analysis plan and (iv) dissemination of results,^5^ and illustrate the extent to which this stakeholder engagement effort achieved the five goals of stakeholder engagement described above.^7^, ^18^ We also describe how we implemented a structured systematic approach to incorporate parent feedback.

## 2 METHODS

### 2.1 Setting: the Padres Efectivos research study

Parent participation in the research process is described in this study in the context of a research project called Padres Efectivos (Parent Activation): Skills Latina Mothers Use to Get Healthcare For Their Children study.^13^ The Padres Efectivos project is a 3‐year research study funded by the PCORI to improve the mental health care and outcomes of Latina/o children with mental health needs. The Padres study tests the comparative effectiveness of an enhanced, culturally sensitive, advocacy skills intervention delivered across four group sessions to build activation among Latina/o families and improve outcomes for their children with mental health needs compared to social support control group. Activation skills are a promising strategy to improve child mental health service use and to bridge cultural differences.^17^ The Institutional Review Board approval for the Padres Efectivos study was obtained from the authors’ institution.

### 2.2 The Padres Efectivos population

Parents who were recruited into the Padres Efectivos research study were self‐identified Latina/o parents who brought their child or dependent ages 2‐22 to receive mental health services in a community‐based clinic located in a southern mid‐size city. Inclusion criteria were child receipt of services with a provider in the clinic at the time of enrolment and consent to participate in the study. Exclusion criteria were the child not residing with the parent and study team concern about behaviour constituting danger to self or others. The partner clinic serves Latina/o immigrant families from low‐income and Spanish language dominant backgrounds. Bilingual, culturally informed evidence‐based behavioural and pharmacological treatment is provided to adults and children presenting with a range of mental health needs. Bilingual and bicultural counsellors, therapists and psychiatrists provide mental health services to children, youth and adults.

### 2.3 The mentor parent advisory group

Researchers facilitated a regular collaboration with parents participating in the research study by establishing an on‐going mentor parent advisory group early in the study. The goal of the mentor parent group is to provide opportunities for parents to discuss research protocols, provide input into study implementation decisions, interpret findings and suggest next steps. Soon after the Padres project received funding from PCORI, the mentor parent group convened and met every three months to discuss various aspects of the research study. The first mentor parent who joined the group had participated months earlier in a parent focus group to inform the Padres study proposal.

Once the trial was underway, the study team facilitators and the project coordinator identified parent study participants from whom we had completed data collection, who showed leadership, meeting commitment and who were interested in giving back to the community by providing feedback about their study group sessions and about our research efforts. The goal was to include parents who had had the experience of participating in the study so that they could provide informed input on the experience of participating and interpreting data. The mentor parent group grew over time, with group size ranging from one to seven parents, and with an average attendance of four parents per meeting.

### 2.4 Compensation

Study participants were paid twenty dollars for each point of data collection. Mentor parents were considered study participants and paid twenty dollars for attendance at each group meeting. Refreshments and childcare were provided at each parent group meeting.

### 2.5 Case study methodology

This study uses a descriptive case study approach to describe the structure, process and outcomes of the Padres mentor parent group in the context of a PCORI‐funded CER study. We refer to those parents who participated in the Padres Efectivos trial as study participants. We refer to those parents who also participated as collaborators with the research team as mentor parents’ advisors.

Mentor parents’ concrete experiences and contributions to the study are described for each of the following stages of the research process following Mullins.^5^

1. Proposal development: Mentor parents’ contributions to design of the study arms.
2. Implementation of study protocols: Mentor parents’ contribution to the best approach and recruitment strategies to collect information from families to meet recruitment goals and minimize dropouts.
3. Analysis plan: Mentor parents’ conceptualization of their parenting role, maturation in that role, the context for help‐seeking on behalf of their child, and implications for the relevance of selected variables and factors to be measured in the study and variables that were not documented in the literature.
4. Dissemination of results: Parents’ review of results to assure believability, relevance and to help other parents and community members understand the information.

Each of these stages in which mentor parents collaborated with researchers had different purposes and particular implications for time, resources and subsequent research activities. Researchers spearheaded this evaluation of the mentor parents’ contributions to the research process and parents provided feedback and recommendations during quarterly meetings.

The mentor parent group sessions were held at the community‐based clinic partnering with academic researchers for the Padres Efectivos project. The group had a structured agenda for each of the sessions to discuss study goals, recruitment, data collection protocols, study measures and analytic methods. As study findings became available, the group met to review those findings and to provide recommendations for interpretation, synthesis and plans for dissemination. The mentor parent feedback pertinent to the partner clinic was also shared with them in the form of written reports and personal communications.

Parents were very willing to participate and share ideas after a foundation of mutual understanding, and trust was built. For example, we scheduled meeting times after consulting with parents and prioritizing the times that worked with their schedules. We always invited a few more participants than we sought to account for unexpected events and typical attrition, provided childcare and food to make attendance more feasible for busy families and provided time and support for parents to warm‐up to each other. We also fostered parent sharing of their opinions in a group setting by having informal discussion over snacks, discussing expectations of the group at the onset, and modelling listening attentively and sharing ideas.

The mentor parent group sessions were conducted in Spanish because it was selected by parents as the language in which they felt most comfortable expressing their ideas. Each session was led by bilingual group coordinators who were also research team members. Sessions were audio recorded following the parents’ oral consent, transcribed and translated into English by a native Spanish speaker. One additional bilingual team member reviewed each transcript while listening to each recording to assure accuracy. Notes taken by the group coordinators as well as a summary of each transcribed session were shared with the entire research team within a week after each parent meeting to establish an on‐going feedback loop where feedback from the mentor parents was promptly shared and discussed with the research team. In turn, decisions made by the research team based on the mentor parents’ feedback was shared and discussed with the parents during their meetings. We conducted member checking, sharing findings and our interpretation of them with parents to confirm that we had captured their data correctly and to maintain transparency in all phases of the research.^20^

The goal of the parent meetings was to for them to provide advice and guidance to the researchers to improve the Padres Efectivos study. In addition to obtaining guidance from mentor parents through the formal mentor parent group, the study team also held study participant focus groups, a staff meeting at the clinic, a community encounter meeting and quarterly meetings with clinic leadership to incorporate other stakeholders’ input throughout the research process. This study focuses on the mentor parent group contributions to the study.

## 3 RESULTS

Table 1 describes the mentor parent contributions at each stage of the CER research process.

**Table 1:** Description of mentor parent contributions to the study at each stage

Stage in CER Process	Mentor parent recommendations and resulting changes in the Padres project
Proposal development	*Timing*: Parents suggested that the intervention be delivered at the start of their child's treatment because it would help build a sense of support and lessen their feelings of isolation early on. They also recommended a length of four sessions. *Study arm structure*: Parents explained that having a group intervention was preferable because they valued the support of other parents experiencing similar situations. *Intervention content*: Parents suggested focusing on improving communication between parents and children. Each of these recommendations was incorporated in the study.
Implementation of study protocols	*Recruitment and retention*: Mentor parents told us that participants felt they could not continue participating if they missed a group session or their child stopped coming to the clinic. In turn, we have emphasized to parents that they are welcome to continue in the study even in the case of these events. As a result, among the first 16 cohorts, 72% of participants attended at least three of four group sessions, and there has been a 3% dropout rate after an individual has been assigned a parent group.
Analysis plan	*Measures*: (i) The mentor parents explained that school success is an important factor motivating parents to seek mental health services for their child. We added two measures to capture confidence in and quality of parent interactions with school personnel, (ii) the mentor parents expressed concern that parents who are new to the health‐care system are very different in terms of their confidence and skills when interacting with providers compared to parents with previous contacts. As a result, we added two measures to assess if a participant had previous experience with the mental health system and where, (iii) mentor parents discussed how it would be easy to convey a high level of activation out of enthusiasm without actually having the skills to accomplish the visit goals. This discussion underscored the appropriateness of the study's use of alternative measures of parent activation: self‐report, clinical encounter and archival record review. *Data analysis*: Mentor parents emphasized the need to account for differences in the level of confidence that parents have at the beginning of the project during the data analysis process and as a way to contextualize the data. This feedback underscored the importance of our planned statistical analyses.
Dissemination of Results	The results of the Padres Efectivos study have been disseminated in the following ways: *Written report to the partner clinic*: A one‐page written report with mentor parent comments on the services provided by the clinic and on their experiences as members of the study mentor parent group that was shared with the clinic director and clinical manager. The report included direct quotes from parents, and this information was shared with therapists and staff at the clinic as well and uploaded to the clinic's website. *Dissemination of findings to outside audiences*: Mentor parents expressed how important it was to help other parents and community members understand the information gathered by the researchers as well as with researchers. They particularly mentioned the importance of implementing findings in school settings. Dissemination efforts are on‐going, including piloting the intervention in schools.

### 3.1 Recommendations and impacts during proposal development

**Recommendation** Based on the feedback provided by clinic parent clientele during the two focus groups organized at the clinic during proposal development, we made the following changes to the study. Parents suggested focusing on improving communication between parents and children. This content area was then incorporated in the first curriculum session of the intervention. Focus group parents explained that having a group intervention was preferable because they valued the support of other parents experiencing similar situations. They also suggested that the intervention be delivered at the start of their child's treatment because it would help build a sense of support and lessen their feelings of isolation early on. We asked focus group and mentor parents about the preferred number of sessions and they agreed that a short intervention was preferable. As a result, we implemented four group sessions early on in the clinical process when families had just finished their initial evaluation at the clinic or when they were placed on a waiting list. **Impact** Each of these recommendations was incorporated in the study.

### 3.2 Recommendations and impacts on study protocols

#### 3.2.1 Recruitment and retention

**Recommendation** The first mentor parent provided feedback that every parent recruited should be included in the numbers, even if they have not been in a group yet. She also recommended that we report how many parents attended at least three of four meetings. **Impact** This echoed and enhanced the credibility of our planned intent‐to‐treat analysis.

**Recommendation** The mentor parents provided important insights on language. They emphasized the importance of the language we use. The mentor parents stressed that the language that health‐care service providers, group facilitators and researchers use with parents should be simple, easy to understand and without medical terminology or jargon. Use of simple language avoids raising fears among caregivers and helps increase their trust, especially as being involved with the mental health care system may be new for many. One parent described an experience that a friend had with a mental health service provider: *“…and the doctor told [the parent] that the child had [name of the diagnosis] and she thought the child was going to die, and she was nervous the entire session.”* **Impact** This made us aware that common ways of communicating diagnoses can be frightening to many parents and reminded us to avoid overly technical terms and jargon to be transparent.

**Recommendation** Once the study was implemented, our mentor parents expressed concern that participants felt they had to drop out if they missed a session. **Impact** The project coordinator began to emphasize to each parent at each contact that they were welcome to continue participating in the groups even if they missed a session or if their child was no longer receiving services at the partner clinic. Upon implementation of this strategy, recruitment and retention were significantly improved. As shown in Table 1, attendance at sessions was high for both intervention and control groups (92%).

### 3.3 Recommendations and impacts regarding analysis plan

#### 3.3.1 Measures

**Figure 1:**
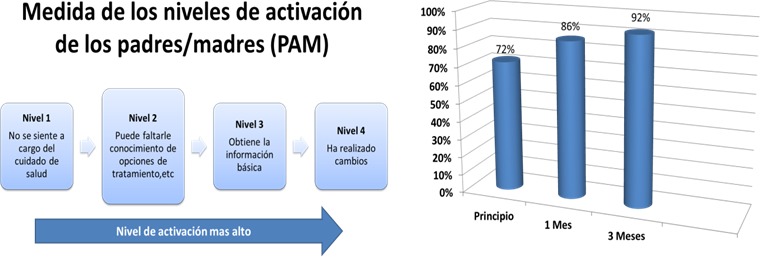
Visual aids created by researchers to convey research data to mentor parents

**Recommendation** The relevance of the primary outcome for the study—parent activation—was discussed with the mentor parents in one of their meetings. The Patient Activation Measure (PAM) was selected to capture parent activation within a health‐care setting on behalf of their child.^16^ A visual presentation of the PAM was provided to the mentor parents along with an explanation of the type of data that measure was intended to collect (See Figure 1). Mentor parents listened attentively, asked questions and confirmed the importance of the PAM as a way to gather information on the parents’ ability to advocate for their children's needs. **Impact** The discussion validated our primary outcome measure as central to parents’ conceptual model of help‐seeking and advocating on behalf of their child.

**Recommendation** Mentor parents also recommended that a measure of parent activation in a school setting be included, particularly regarding the parent‐teacher relationship. **Impact** As a result, two additional measures were included early on in the study—an exploratory measure of school activation and the Parent‐Teacher Involvement Scale.^21^ These tools were designed to measure parent activation in a school context and the quality of the relationship with teachers, respectively. Quantitative data analyses of these school measures showed an increase over time in both groups of parents, intervention and control. This change was greater for the intervention group.^22^ Importantly, in interim reports to the funder, the school measures showed an impact of the intervention before the PAM. The strong impact on these measures underscores their relevance for parents.

#### 3.3.2 Data analysis

**Recommendation** Parents also expressed concern that the Parent Activation Measure (PAM) might not be capturing parents’ constant need for learning that exists along with their high levels of confidence. Mentor parents felt that there is always a need for growth and constant learning on how to best interact and advocate for their child's services. One parent explained it this way: “*… I always think…although I feel very active [i.e., activated] and with all of the information that I need, and you know, made a lot of changes in the house, still I always feel there is something that I couldn't learn and I that I could improve…”* **Impact** This distinction between perceptions of self‐efficacy and actual advocacy skills that lead to desired outcomes is critical. The Padres study was designed to address this distinction by measuring study outcomes from three sources: parent report (to capture perceptions), visit audio tape (to capture content of parent‐provider communication) and chart abstraction (to capture child service use over time). The mentor parent discussion underscored the relevance of our set of measures.

**Recommendation** The mentor parents explained that parents who were already involved with the mental health system would be very different in their level of activation and readiness to participate in the group and use the learned skills from parents who were new to services at the partner clinic. Mentor parents suggested adding a measure to account for these potential differences. **Impact** Differences among parents participating in the treatment and control groups at baseline were ideally accounted for through randomization. Nonetheless, based on this mentor parent recommendation, two items were added to account for their conceptual model of activation and to assess whether the participant received mental health treatment during the past year and whether the treatment was at the study clinic or elsewhere.

**Recommendation** During mentor parent discussion of the importance of fairly comparing parents who are participating in the groups, they stressed the need to account for differences in the level of confidence that parents have at the beginning of the project when comparing outcomes between the groups. **Impact** Their framework for understanding growth in activation over time underscored the importance of our randomized controlled trial with difference‐in‐difference analytical approach.

### 3.4 Recommendations and impacts regarding dissemination of results

**Figure 2:**
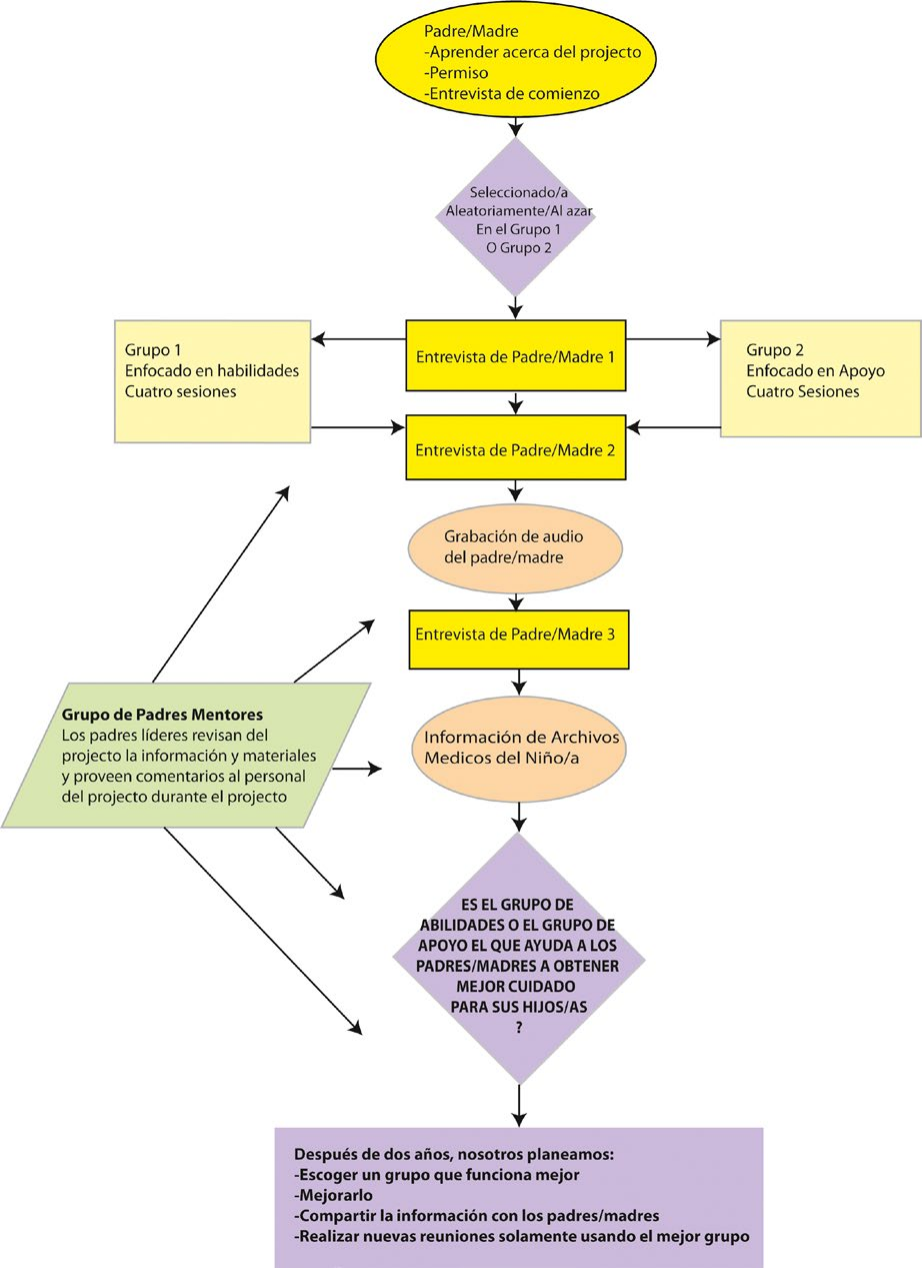
Visual aid to convey the research study design to parents and other audiences

**Recommendation** The mentor parents suggested the use of visual aids to better convey research concepts to them and as a means to facilitate accurate comprehension of the Padres Efectivos research design during the dissemination process (See Figure 2). They emphasized the importance of conveying the big picture of the study design through a flow chart and discussed details of the group contact important to them as well. **Impact** Researchers used visuals to facilitate subsequent mentor parent discussion. The chart developed by the mentor parents was also used in presentations to researchers.

#### 3.4.1 Dissemination of findings to outside audiences

**Recommendation** Towards the end of the study researchers discussed with mentor parents strategies to disseminate study findings to relevant audiences. The mentor parents expressed how important it was to help other parents and community members understand the information gathered by the researchers. For example, mentor parents wanted to share the intervention with schools and community members in an open and informal type of setting. One mentor parent stated this research priority this way: “I think it would be really good…there are many ways to reach people, I think it is easier if they hear it from someone like…I mean not so much from researchers and doctors…they are important but when they [groups learning about our research findings] see a “real person”, is saying it from the “how it worked for them” is much more…like it has more influence. Like saying “no, look, it works and she [mentor parents, along with the research team, disseminating study results to the community] is here saying that [the study] it helped and how it helped. [Translated from Spanish]” In addition, mentor parents expressed interest in presenting the study results in front of individual donors affiliated with the partner clinic. **Impact** Dissemination of study findings is on‐going, in response to parent suggestions.

**Recommendation** Mentor parents also felt it was important to share the intervention and their feedback broadly. They recommended sharing quotes about their experiences with families who may be ambivalent about seeking services as well as schools, community members, and potential benefactors and clinic supporters. They value using the intervention as a “vaccine” to prevent child mental health needs. **Impact** The mentor parents collaborated in the preparation of this manuscript confirming the descriptions of their involvement and impacts in the research study process and by suggesting the inclusion of direct quotes from parents and figures with appealing colour visuals.

**Recommendation** Mentor parents suggested implementing the Padres Efectivos intervention in schools given the relevance of this context in facilitating mental health services for their children. **Impact** We were not able to directly implement this recommendation given funding constraints. We discussed with parents this suggestion and our limitations, and we informed them of our efforts to seek funding to expand this project in a school setting as part of our sustainability efforts. This school‐based project is currently underway as a pilot study, and parents were informed of this development and updated on its progress.

## 4 DISCUSSION

The input from mentor parents was part of an on‐going dialogue with researchers throughout the research study where mentor parents helped the research team guide questions, consider future directions and contextualize the emerging data. This study describes the concrete contributions of Latina/o mentor parents to study design, implementation and outcomes in the context of a CER study. The authors acknowledge that several factors contributed to the quality of the Padres Efectivos research study in these areas. Mentor parent collaboration leads to important improvements in study design, implementation protocols and ultimately to measurement of study impacts.

The advice provided by mentor parents helped overcome barriers for other parents to participate in the research study by helping researchers understand that some parents may have felt uncomfortable or “apenado/a” (embarrassed) returning to the study after missing one group session. We learned that a simple statement made by the group facilitator to encourage parents to continue participating in the study, even if they had missed one session, contributed to parents’ willingness to return. In fact, the Padres Efectivos study had a very low study participant dropout rate over three months for this population (27%).

Meaningful parent participation was also facilitated by researchers’ efforts. Those efforts include the development of an infrastructure to elicit feedback, on‐going communication and allocation of resources such as incentives and a bilingual researcher leading this effort.^8^ These practices have been deemed best practices for good research collaborations. These practices have been deemed best practices for good research collaborations.^23^ For example, having an on‐going group meeting at the partner clinic's conference room to incorporate parent voices in the research process positively influenced the implementation of study protocols, analysis plan, data collection and dissemination of results. The importance of parent engagement as research collaborators was directly expressed by a mentor parent as follows: “To want to help in what you can. And if with one's opinion someone else can get help, I say that is what motivates us to be here [at a mentor parent group meeting], right? That thinking that something that we can say or do here can help other parents, can help other people who are talking to these parents to do it in a better way to help our children.” [Translated from Spanish]. This finding is consistent with past literature documenting that successful partnerships between Latino families and community settings are most effective when families are provided opportunities for community advocacy, and researchers engage families in equal partnerships.^24^

Based on the mentor parent contributions to this research study, we learned that activation is a fluid construct that intersects clinical and educational domains, which were the top priority settings for this population. We knew from the literature that education had a special significance to Latina/o families.^25^, ^26^ However, we learned that it may be the first setting where the first signs of changes in parent activation may be detected. Additional studies should adapt and test parent activation measures on this intercontextual influence. In addition, the concept of a parent new to the mental health care system vs a more experienced parent as it related to parent activation takes on a unique connotation for this population. That is, some novice parents may include recent immigrants from Latin American countries who are likely to have different expectations, values, assigned roles as patients, for example a strong cultural deference to authority, and experiences with health‐care services from their native country. This finding is in line with past work that documents that sociocultural and acculturative factors influence access to mental health care in Latino populations.^27^ Thus, research efforts seeking to elucidate the impact of parent activation training for this population should account for these subgroup differences.

There were limitations in this parent involvement process that are worth further discussion. First, these results may not be generalizable to other communities. That is, aspects of parent activation that were relevant for mentor parents in the context of this study may be different for non‐immigrant communities. Second, it was difficult for mentor parents to fully participate in dissemination efforts as intended by a participatory research approach. For example, researchers obtained funding for one mentor parent to attend a national public health conference to present study findings along with the research team. However, the mentor parents were not able to travel a long distance to attend a national conference because of competing responsibilities. The importance of planning such participation in advance at outlets that fit participants’ needs became apparent (e.g. regional conferences and events, use of teleconferencing or prerecorded presentations). In addition to the benefits of inclusion in research efforts on communities, awareness of participants’ needs and the burdens of engaging in research should be a priority.

The mentor parents’ advice has contributed to the project's recruitment goals that have exceeded expectations. In addition, their insights on measurement issues have enriched study data and improved researcher's ability to document valued study outcomes. Overall, these findings can inform future research efforts seeking an active Latina/o parent collaboration and the timely incorporation of parent voices in each phase of the research process.

## CONFLICT OF INTEREST

No conflicts to report.
